# GENCODE 2021

**DOI:** 10.1093/nar/gkaa1087

**Published:** 2020-12-03

**Authors:** Adam Frankish, Mark Diekhans, Irwin Jungreis, Julien Lagarde, Jane E Loveland, Jonathan M Mudge, Cristina Sisu, James C Wright, Joel Armstrong, If Barnes, Andrew Berry, Alexandra Bignell, Carles Boix, Silvia Carbonell Sala, Fiona Cunningham, Tomás Di Domenico, Sarah Donaldson, Ian T Fiddes, Carlos García Girón, Jose Manuel Gonzalez, Tiago Grego, Matthew Hardy, Thibaut Hourlier, Kevin L Howe, Toby Hunt, Osagie G Izuogu, Rory Johnson, Fergal J Martin, Laura Martínez, Shamika Mohanan, Paul Muir, Fabio C P Navarro, Anne Parker, Baikang Pei, Fernando Pozo, Ferriol Calvet Riera, Magali Ruffier, Bianca M Schmitt, Eloise Stapleton, Marie-Marthe Suner, Irina Sycheva, Barbara Uszczynska-Ratajczak, Maxim Y Wolf, Jinuri Xu, Yucheng T Yang, Andrew Yates, Daniel Zerbino, Yan Zhang, Jyoti S Choudhary, Mark Gerstein, Roderic Guigó, Tim J P Hubbard, Manolis Kellis, Benedict Paten, Michael L Tress, Paul Flicek

**Affiliations:** European Molecular Biology Laboratory, European Bioinformatics Institute, Wellcome Genome Campus, Hinxton, Cambridge CB10 1SD, UK; UC Santa Cruz Genomics Institute, University of California, Santa Cruz, Santa Cruz, CA 95064, USA; MIT Computer Science and Artificial Intelligence Laboratory, 32 Vassar St, Cambridge, MA 02139, USA; Broad Institute of MIT and Harvard, 415 Main Street, Cambridge, MA 02142, USA; Centre for Genomic Regulation (CRG), The Barcelona Institute for Science and Technology, Dr. Aiguader 88, Barcelona, E-08003 Catalonia, Spain; European Molecular Biology Laboratory, European Bioinformatics Institute, Wellcome Genome Campus, Hinxton, Cambridge CB10 1SD, UK; European Molecular Biology Laboratory, European Bioinformatics Institute, Wellcome Genome Campus, Hinxton, Cambridge CB10 1SD, UK; Department of Molecular Biophysics and Biochemistry, Yale University, New Haven, CT 06520, USA; Department of Bioscience, Brunel University London, Uxbridge UB8 3PH, UK; Functional Proteomics, Division of Cancer Biology, Institute of Cancer Research, 237 Fulham Road, London SW3 6JB, UK; UC Santa Cruz Genomics Institute, University of California, Santa Cruz, Santa Cruz, CA 95064, USA; European Molecular Biology Laboratory, European Bioinformatics Institute, Wellcome Genome Campus, Hinxton, Cambridge CB10 1SD, UK; European Molecular Biology Laboratory, European Bioinformatics Institute, Wellcome Genome Campus, Hinxton, Cambridge CB10 1SD, UK; European Molecular Biology Laboratory, European Bioinformatics Institute, Wellcome Genome Campus, Hinxton, Cambridge CB10 1SD, UK; MIT Computer Science and Artificial Intelligence Laboratory, 32 Vassar St, Cambridge, MA 02139, USA; Broad Institute of MIT and Harvard, 415 Main Street, Cambridge, MA 02142, USA; Computational and Systems Biology Program, Massachusetts Institute of Technology, Cambridge, MA, USA; Centre for Genomic Regulation (CRG), The Barcelona Institute for Science and Technology, Dr. Aiguader 88, Barcelona, E-08003 Catalonia, Spain; European Molecular Biology Laboratory, European Bioinformatics Institute, Wellcome Genome Campus, Hinxton, Cambridge CB10 1SD, UK; Bioinformatics Unit, Spanish National Cancer Research Centre (CNIO), Madrid, Spain; European Molecular Biology Laboratory, European Bioinformatics Institute, Wellcome Genome Campus, Hinxton, Cambridge CB10 1SD, UK; UC Santa Cruz Genomics Institute, University of California, Santa Cruz, Santa Cruz, CA 95064, USA; European Molecular Biology Laboratory, European Bioinformatics Institute, Wellcome Genome Campus, Hinxton, Cambridge CB10 1SD, UK; European Molecular Biology Laboratory, European Bioinformatics Institute, Wellcome Genome Campus, Hinxton, Cambridge CB10 1SD, UK; European Molecular Biology Laboratory, European Bioinformatics Institute, Wellcome Genome Campus, Hinxton, Cambridge CB10 1SD, UK; European Molecular Biology Laboratory, European Bioinformatics Institute, Wellcome Genome Campus, Hinxton, Cambridge CB10 1SD, UK; European Molecular Biology Laboratory, European Bioinformatics Institute, Wellcome Genome Campus, Hinxton, Cambridge CB10 1SD, UK; European Molecular Biology Laboratory, European Bioinformatics Institute, Wellcome Genome Campus, Hinxton, Cambridge CB10 1SD, UK; European Molecular Biology Laboratory, European Bioinformatics Institute, Wellcome Genome Campus, Hinxton, Cambridge CB10 1SD, UK; European Molecular Biology Laboratory, European Bioinformatics Institute, Wellcome Genome Campus, Hinxton, Cambridge CB10 1SD, UK; Department of Medical Oncology, Inselspital, University Hospital, University of Bern, Bern, Switzerland; Department of Biomedical Research (DBMR), University of Bern, Bern, Switzerland; European Molecular Biology Laboratory, European Bioinformatics Institute, Wellcome Genome Campus, Hinxton, Cambridge CB10 1SD, UK; Bioinformatics Unit, Spanish National Cancer Research Centre (CNIO), Madrid, Spain; European Molecular Biology Laboratory, European Bioinformatics Institute, Wellcome Genome Campus, Hinxton, Cambridge CB10 1SD, UK; Department of Molecular, Cellular & Developmental Biology, Yale University, New Haven, CT 06520, USA; Systems Biology Institute, Yale University, West Haven, CT 06516, USA; Department of Molecular Biophysics and Biochemistry, Yale University, New Haven, CT 06520, USA; European Molecular Biology Laboratory, European Bioinformatics Institute, Wellcome Genome Campus, Hinxton, Cambridge CB10 1SD, UK; Department of Molecular Biophysics and Biochemistry, Yale University, New Haven, CT 06520, USA; Bioinformatics Unit, Spanish National Cancer Research Centre (CNIO), Madrid, Spain; European Molecular Biology Laboratory, European Bioinformatics Institute, Wellcome Genome Campus, Hinxton, Cambridge CB10 1SD, UK; European Molecular Biology Laboratory, European Bioinformatics Institute, Wellcome Genome Campus, Hinxton, Cambridge CB10 1SD, UK; European Molecular Biology Laboratory, European Bioinformatics Institute, Wellcome Genome Campus, Hinxton, Cambridge CB10 1SD, UK; European Molecular Biology Laboratory, European Bioinformatics Institute, Wellcome Genome Campus, Hinxton, Cambridge CB10 1SD, UK; European Molecular Biology Laboratory, European Bioinformatics Institute, Wellcome Genome Campus, Hinxton, Cambridge CB10 1SD, UK; European Molecular Biology Laboratory, European Bioinformatics Institute, Wellcome Genome Campus, Hinxton, Cambridge CB10 1SD, UK; Centre of New Technologies, University of Warsaw, Warsaw, Poland; Department of Biomedical Informatics at Harvard Medical School, 10 Shattuck Street, Suite 514, Boston, MA 02115, USA; Department of Molecular Biophysics and Biochemistry, Yale University, New Haven, CT 06520, USA; Department of Molecular Biophysics and Biochemistry, Yale University, New Haven, CT 06520, USA; Program in Computational Biology & Bioinformatics, Yale University, Bass 432, 266 Whitney Avenue, New Haven, CT 06520, USA; European Molecular Biology Laboratory, European Bioinformatics Institute, Wellcome Genome Campus, Hinxton, Cambridge CB10 1SD, UK; European Molecular Biology Laboratory, European Bioinformatics Institute, Wellcome Genome Campus, Hinxton, Cambridge CB10 1SD, UK; Department of Molecular Biophysics and Biochemistry, Yale University, New Haven, CT 06520, USA; Department of Biomedical Informatics, College of Medicine, The Ohio State University, Columbus, OH 43210, USA; Functional Proteomics, Division of Cancer Biology, Institute of Cancer Research, 237 Fulham Road, London SW3 6JB, UK; Department of Molecular Biophysics and Biochemistry, Yale University, New Haven, CT 06520, USA; Program in Computational Biology & Bioinformatics, Yale University, Bass 432, 266 Whitney Avenue, New Haven, CT 06520, USA; Department of Computer Science, Yale University, Bass 432, 266 Whitney Avenue, New Haven, CT 06520, USA; Centre for Genomic Regulation (CRG), The Barcelona Institute for Science and Technology, Dr. Aiguader 88, Barcelona, E-08003 Catalonia, Spain; Universitat Pompeu Fabra (UPF), Barcelona, E-08003 Catalonia, Spain; Department of Medical and Molecular Genetics, King's College London, Guys Hospital, Great Maze Pond, London SE1 9RT, UK; MIT Computer Science and Artificial Intelligence Laboratory, 32 Vassar St, Cambridge, MA 02139, USA; Broad Institute of MIT and Harvard, 415 Main Street, Cambridge, MA 02142, USA; UC Santa Cruz Genomics Institute, University of California, Santa Cruz, Santa Cruz, CA 95064, USA; Bioinformatics Unit, Spanish National Cancer Research Centre (CNIO), Madrid, Spain; European Molecular Biology Laboratory, European Bioinformatics Institute, Wellcome Genome Campus, Hinxton, Cambridge CB10 1SD, UK

## Abstract

The GENCODE project annotates human and mouse genes and transcripts supported by experimental data with high accuracy, providing a foundational resource that supports genome biology and clinical genomics. GENCODE annotation processes make use of primary data and bioinformatic tools and analysis generated both within the consortium and externally to support the creation of transcript structures and the determination of their function. Here, we present improvements to our annotation infrastructure, bioinformatics tools, and analysis, and the advances they support in the annotation of the human and mouse genomes including: the completion of first pass manual annotation for the mouse reference genome; targeted improvements to the annotation of genes associated with SARS-CoV-2 infection; collaborative projects to achieve convergence across reference annotation databases for the annotation of human and mouse protein-coding genes; and the first GENCODE manually supervised automated annotation of lncRNAs. Our annotation is accessible via Ensembl, the UCSC Genome Browser and https://www.gencodegenes.org.

## INTRODUCTION

GENCODE produces widely-used reference genome annotation of protein-coding and non-coding loci including alternatively spliced transcripts and pseudogenes for the human and mouse genomes and makes these annotations freely available for the benefit of biomedical research and genome interpretation. The GENCODE consortium develops, maintains and improves targeted tools, analysis and primary transcriptomic and proteomic data in support of gene and transcript annotation. These resources support updates to genes in all functional classes or biotypes, including (i) the discovery of new features such as novel protein-coding genes and long non-coding RNA (lncRNA) genes; (ii) the extension of existing annotation including the identification of novel alternatively spliced transcripts at protein-coding and lncRNA loci and (iii) the continuous critical reappraisal of existing annotation that may result in removal or reclassification of protein-coding genes that lack evidence of protein-coding potential given all data now available. GENCODE defines genes in terms of their transcriptional and functional overlap. The functional information implicit in the CDS of protein-coding gene supports decision making and provides high confidence in the interpretation of protein-coding genes. For lncRNAs, the lack of analogous knowledge makes representation of complex lncRNA loci difficult and we are working with lncRNA community and other reference annotation databases to improve their annotation.

Among other achievements, over the last two years we have developed a manually supervised automated annotation pipeline and an annotation triage tool to leverage the volume of data generated by current transcriptomics experiments while ensuring that the resulting annotated transcript models maintain the quality of expert human annotation. We have completed the first pass manual annotation of the mouse reference genome based on experiences on completing the human annotation in 2013 and have used whole genome PhyloCSF (1) analysis to generate ranked lists of candidate coding regions for investigation by expert human annotators. To support research responding to the COVID-19 pandemic, we have reviewed and improved the annotation for a set of protein-coding genes associated with SARS-CoV-2 infection and immediately released the results using a trackhub (2). We worked with the RefSeq (3) and UniProt (4) reference annotation databases toward achieving annotation convergence by ensuring that when a protein-coding gene or protein is present in one resource, it will be represented in the others or there will be an explanation why not. We are part of the Matched Annotation from NCBI and EMBL-EBI (MANE) project to define a single representative ‘MANE Select’ transcript for all protein-coding genes and ensure its structure and sequence is identical in both the Ensembl/GENCODE and RefSeq genesets. We annotated new human protein-coding genes based on improved analyses and experimental validation using mass spectrometry. We have also improved the annotation of lncRNAs via the discovery of novel loci and novel transcripts at existing loci primarily based on incorporating long transcriptomic sequence data generated using the CLS protocol (5).

## GENE ANNOTATION INFRASTRUCTURE

We have made several key improvements to our processes and tools used for manual gene annotation.

The Ensembl/GENCODE geneset is a merge of the manual gene annotation created by the Ensembl-HAVANA team (methods and validation described in 6–8) and the automated annotation produced by the Ensembl Genebuild team (9,10). Historically, these data were produced separately and stored in independent and structurally different databases before being merged into a single set for release. To speed data release and reduce complexity, we have now moved all manual annotation and computational annotation into a single database for human (and another for mouse). In addition to continuing the support of manual annotation, this transition allows manual annotators to directly ‘bless’, update or remove computationally annotated models. Most significantly, new genes and transcripts released early via the GENCODE update trackhub will be assigned their Ensembl (ENSX) formatted stable IDs at their creation, having previously been given an interim ID (OTTX format).

**Figure 1.**
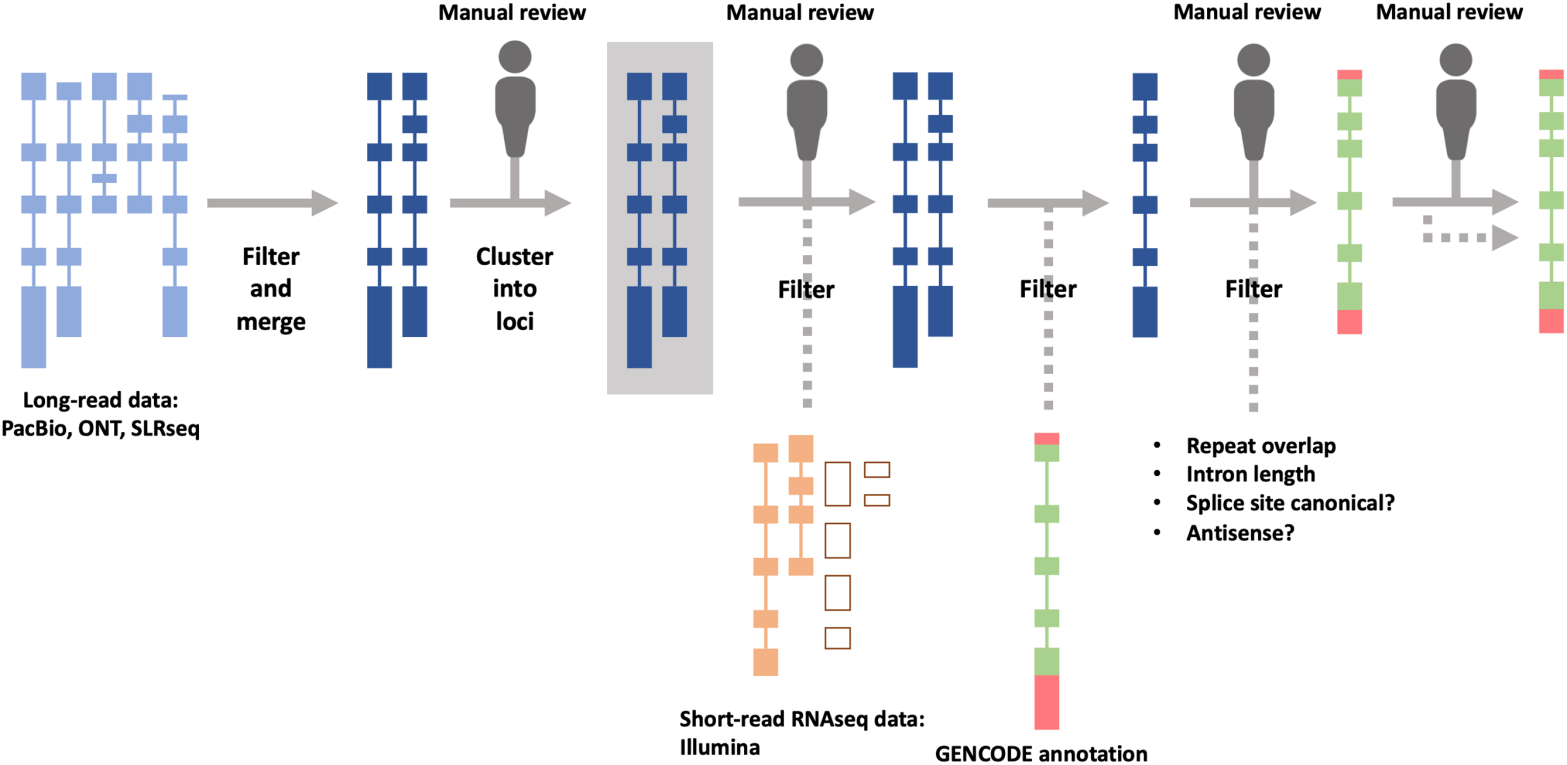
Schematic of the TAGENE workflow to add long transcriptomic data to GENCODE annotation. Points in the workflow where manual review is applied are indicated.

Long-read transcriptomic sequencing methods including those from Pacific Biosciences (PacBio) and Oxford Nanopore Technologies (ONT) produce data volumes that require change to our manual annotation process. In response, we developed the TAGENE pipeline to support greater automation of transcript model creation based on long-read datasets generated both within GENCODE and by other groups. TAGENE implements filtering and merging of long transcriptomic datasets before clustering putative transcripts into loci (both existing and novel) and applying further filters based on other transcriptomic datasets, including RNA-seq supported introns and existing GENCODE annotation (Figure 1). The clustering and final filtering steps are applied following multiple iterations of manual review until a point is reached where the false positive rate for the addition of spurious models is <0.1%.

To support higher throughput manual annotation we have developed a web-based gene annotation triage tool (Kestrel; manuscript in preparation). This software allows manual annotators to rapidly visualise, browse through and, via connection to the Annotrack annotation issue-tracking database (11), record decisions for large numbers of gene annotations and QC flags. It has been specifically designed in mind for ‘quick decision’ cases such as high throughput checking the validity of transcript models created by the TAGENE pipeline. Kestrel is complementary to our set of high quality annotation tools in Zmap, Blixem and Dotter, which were initially developed for the clone-by-clone annotation approach used for the first pass annotation of the human and mouse reference genomes. Kestrel's streamlined functionality is often all that is required to answer emerging manual annotation questions and thus faster than our traditional workflow.

## GENE ANNOTATION UPDATES

The GENCODE consortium has improved and extended the annotation of the human and mouse reference genomes and makes the annotation publicly available (see Table 1 for annotation statistics from the most recent GENCODE releases).

**Table 1.** Total numbers of genes and transcripts in the GENCODE 35 (Human) and GENCODE M25 (Mouse) releases by gene functional biotype

			Protein-coding	LncRNA	Pseudogene	sRNA	IG/TR
**Human**	**GENCODE 35**	**Genes**	19954	17957	14767	7569	645
		**Transcripts**	154580	48684	18664	7569	666
**Mouse**	**GENCODE M25**	**Genes**	21859	13197	13741	6108	700
		**Transcripts**	102241	18856	14522	6108	864

Since June 2018, ∼37 000 genes (∼32 000 human and 5000 mouse) and ∼63 000 transcripts (∼55 000 human and ∼8000 mouse) have either been created or updated in the GENCODE geneset (see Table 2 for a breakdown of new and updated genes and transcripts by functional biotype). During this period we have completed the first pass annotation of the mouse reference genome and conducted a number of tightly focussed annotation projects including the human and mouse olfactory receptor repertoire (12) and a re-annotation of developmental and epileptic encephalopathy-associated genes (13).

**Table 2.** Numbers of genes and transcripts that have been added to or updated in GENCODE Human and Mouse annotation since June 2018

		Human			Mouse		
		New	Updated	New and updated	New	Updated	New and updated
	**Protein-coding**	131	17995	18126	845	1584	2429
**Genes**	**LncRNA**	1965	7678	9643	670	282	952
	**Pseudogene**	75	4152	4227	676	266	942
	**Total**	2171	29825	31996	2191	2132	4323
	**Protein-coding**	11334	21406	32740	4323	968	5291
**Transcripts**	**LncRNA**	19042	2807	21849	1171	73	1244
	**Pseudogene**	247	259	506	794	137	931
	**Total**	30623	24472	55095	6288	1178	7466

Although a number of protein-coding genes in both human and mouse have been added, removed or had their biotype changed over the past two years, the total number of genes is stable. Similarly, the number of pseudogenes of protein-coding genes is broadly stable for human, although our ability to better identify unitary pseudogenes has led to an increase in this specific class. In mouse, an increase in pseudogene count reflects the completion of manual annotation for all chromosomes. LncRNAs continue to show the largest increases in number, particularly in human where our efforts have been concentrated.

## PROTEIN-CODING GENES

In response to the SARS-CoV-2 pandemic, we have applied our annotation resources to human genes with potential links to viral infection and COVID-19 disease primarily by investigating whether existing annotation for these genes can be improved. Our list of genes for reannotation comes from several sources including recently published drug repurposing studies identifying host proteins associated with other related coronaviruses (14) and human proteins found to physically associate with SARS-CoV-2 viral proteins in the cell (15). We also included genes curated by UniProt (4) and the Human Cell Atlas project (16) as well as interferon-stimulated genes with known antiviral activity (17). These efforts added previously unannotated alternatively-spliced transcript models and updated existing GENCODE transcript models, in particular ‘partial’ models that were incomplete at their 5′ and/or 3′ ends that could be extended to full length. All annotation takes advantage of long transcriptomic datasets and RNAseq data that was unavailable at the time of initial annotation. To date we have updated the annotation for 280 genes, adding ∼3700 novel transcripts and updating a further ∼850.

GENCODE has been actively collaborating with other reference annotation databases to try to achieve convergence on the annotation of protein-coding genes in human and mouse. The MANE project aims to create a single agreed transcript for every human protein-coding gene that has a 100% match for sequence and structure (splicing, UTR and CDS) in both the Ensembl/GENCODE and RefSeq (3) annotation sets. The project is driven by two independent pipelines, one from each centre, followed by extensive investigation and discussion by expert human annotators where the pipelines do not agree. The latest release of MANE v0.91, gives an overall coverage of 84% of all protein-coding genes.

We have been working extensively to improve the interoperability of the existing annotations with UniProt. **G**enome **I**ntegration with **F**unc**T**ion and **S**equence (GIFTS) is a joint project between Ensembl and the EMBL-EBI component of the UniProt project and is currently available for human and mouse proteins https://www.ebi.ac.uk/gifts/. GIFTS calculates mappings and pairwise alignments between Ensembl transcripts that have a protein translation with their corresponding UniProt protein entries. Unmapped UniProt proteins are investigated by annotators from both teams and edited where necessary. We have investigated 1044 unmapped human (716) and mouse (328) proteins from UniProt and identified cases where the GENCODE annotation needs to be updated (2 human, 49 mouse), and proteins that appear invalid in their putative genomic context (640 human, 54 mouse).

We continue to analyse publications external to the GENCODE consortium reporting additional protein-coding genes in the light of GENCODE criteria. For example, we examined the novel protein-coding genes reported in the CHESS gene annotation set (18), adding five protein-coding genes, 16 pseudogenes and 37 lncRNAs. A recent survey of heart ORFs (19), has so far resulted in the annotation of 12 additional human protein-coding genes.

**Figure 2.**
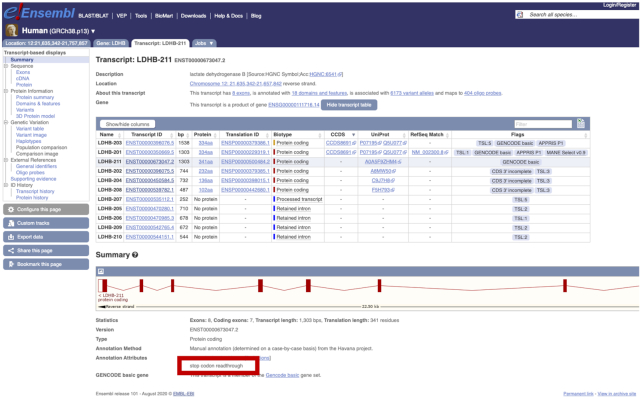
Screenshot from the Ensembl genome browser of the transcript view page for the gene LDHB, which contains a transcript (ENST00000673047, LDHB-211) with an annotated stop-codon readthrough event. The location of the annotation attribute flagging the stop-codon readthrough is highlighted by the red box.

GENCODE annotation makes substantial use of comparative genomics to help identify regions on the genome with protein-coding potential. For example, we have used Cactus to create a 600-way vertebrate whole genome alignment incorporating data from the 200 Mammals and Bird 10K projects as the basis of a single base-pair resolution map of evolutionary selection (20). We will directly use these alignments within the PhyloCSF phylogenetic analysis tool (1). The PhyloCSF pipeline has also been run on the each new release of the human and mouse genome annotations to facilitate the discovery of additional novel coding genes, novel pseudogenes, and novel coding sequence (21). We have automated our process to generate updated lists of PhyloCSF Candidate Coding Regions (PCCRs), which are then examined by manual annotators. In human, PCCRs are part of the standard annotation workflow. In mouse, a targeted review of unannotated PCCRs analogous to that previously undertaken in human has led to the identification of 64 novel protein-coding genes, 376 novel coding exons in preexisting protein-coding genes, and 202 pseudogenes including 56 unitary pseudogenes. PhyloCSF has also been used to identify candidate ribosomal stop codon readthrough events in human and mouse (22,23). Following manual review of these and several others identified experimentally, 14 and 11 genes with stop codon readthrough events have been annotated in human and mouse, respectively (Figure 2).

GENCODE annotation utilises proteomics data to supplement transcriptomic and evolutionary evidence of protein-coding functionality and we have continued to both generate experimental MS data and use publicly available data sets to aid the identification and annotation of protein-coding genes. Our data generation focus is on elements of the proteome that are missed by standard proteomics approaches including the use of 155 novel synthetic peptides targeting distinct and unique peptides mapping to putative coding genes, newly discovered protein coding genes that require validation, and pseudogenes that have shown strong peptide evidence in previous experiments. These peptides are compiled into a reference spectral library, which is used to validate their existence in our experimental proteomics data and large public MS datasets. For example transcriptomic, conservation, and ribosome profiling data combined with experimental peptide evidence supported the discovery and validation of an alternate protein isoform originating from a non-ATG start site in the gene POLG (24), and highlighted a novel class of unannotated protein-coding features that are now under active investigation.

To support the automated analysis of proteomics data for genome annotation we collaborated with the PRIDE (25) proteomics repository at EMBL-EBI to build a reprocessing and peptide-to-genome mapping pipeline for public proteomics.

Finally, we developed a pipeline based on UniProt (4), APPRIS (26), PhyloCSF (1), Ensembl gene trees (10), RNA-seq, MS and variation data to identify annotated protein-coding genes with weak or no support. This method enables us to scrutinise currently annotated protein-coding genes in the human and mouse gene set for misclassified gene models. To date we have flagged as potential non-coding genes more than 2475 human and 1807 mouse genes that were annotated as protein-coding. These are then reviewed in an iterative and ongoing process by expert manual annotators and retained, removed or reclassified based on their current supporting evidence. To date, ∼1000 human protein-coding genes have been reviewed and 119 removed or reclassified. A complementary approach has also been developed to identify missing and partially complete gene models in the human genome and submit to manual review.

## LncRNAS

We have made improvements to the Capture Long Sequencing (CLS) lab protocol (5), including a 5′ cap selection step (‘CapTrap’) (27), which increases the proportion of sequenced full-length transcripts and the use of Spiked-in RNA Variant Control Mixes (SIRVs). Applying CLS, we have generated long transcriptomic data targeting a variety of suspected lncRNA-producing genomic loci in both human and mouse. Focusing primarily on unannotated regions such as GWAS sites, putative enhancers, and non-GENCODE lncRNA catalogs (e.g. miTranscriptome (28), NONCODE (29), FANTOM CAT (30)). In total we have produced more than 36 million ONT reads and 2 million PacBio Sequel (PBS) reads identifying thousands of potential novel loci (∼1600 in Human, ∼4500 in mouse) in currently unannotated genomic regions for review and inclusion in the Ensembl/GENCODE geneset. Long transcriptomic sequence data produced within GENCODE and from public data archives has been run through our TAGENE workflow and the results of this first set of analysis released to the public in GENCODE 31 (June 2019). These initial results have already made a significant difference to the coverage of lncRNAs in GENCODE, with the addition of 1711 novel loci and 17 858 transcripts, an 11% and 60% increase compared to the previous release respectively.

## PSEUDOGENES

Our pseudogene annotation has benefited from the analysis of new datasets. For example, using RNA-seq datasets from ENTEx-pseudogene expression in various human tissues we have developed a computational framework to accurately quantify the expression level of pseudogenes, and identify actively transcribed pseudogenes in each tissue. We have also used our pseudogene annotation in 16 closely related mouse strains from the Mouse Genomes Project (31) to create orthology relationships for the conserved annotations and the identification of patterns of pseudogene gain and loss between strains (32) and give a prototype for work annotating human pseudogenes leveraging variation across the human population.

## DATA ACCESS

GENCODE gene sets are currently updated up to four times each year for both human and mouse. Each release is versioned and made available immediately upon release from Ensembl (6) and https//www.gencodegenes.org with release on the UCSC Genome Browser (33) normally following shortly thereafter. The current human release is GENCODE 35 (August 2020) and the current mouse release is GENCODE M25 (April 2020). Additional information and previous releases can be found at https//www.gencodegenes.org.

GENCODE is the now the standardised default human and mouse annotation for both the Ensembl and UCSC genome browsers following a transition of UCSC’s mouse annotation in April 2019. Data is presented through all of the standard interfaces from both resources.

**Figure 3.**
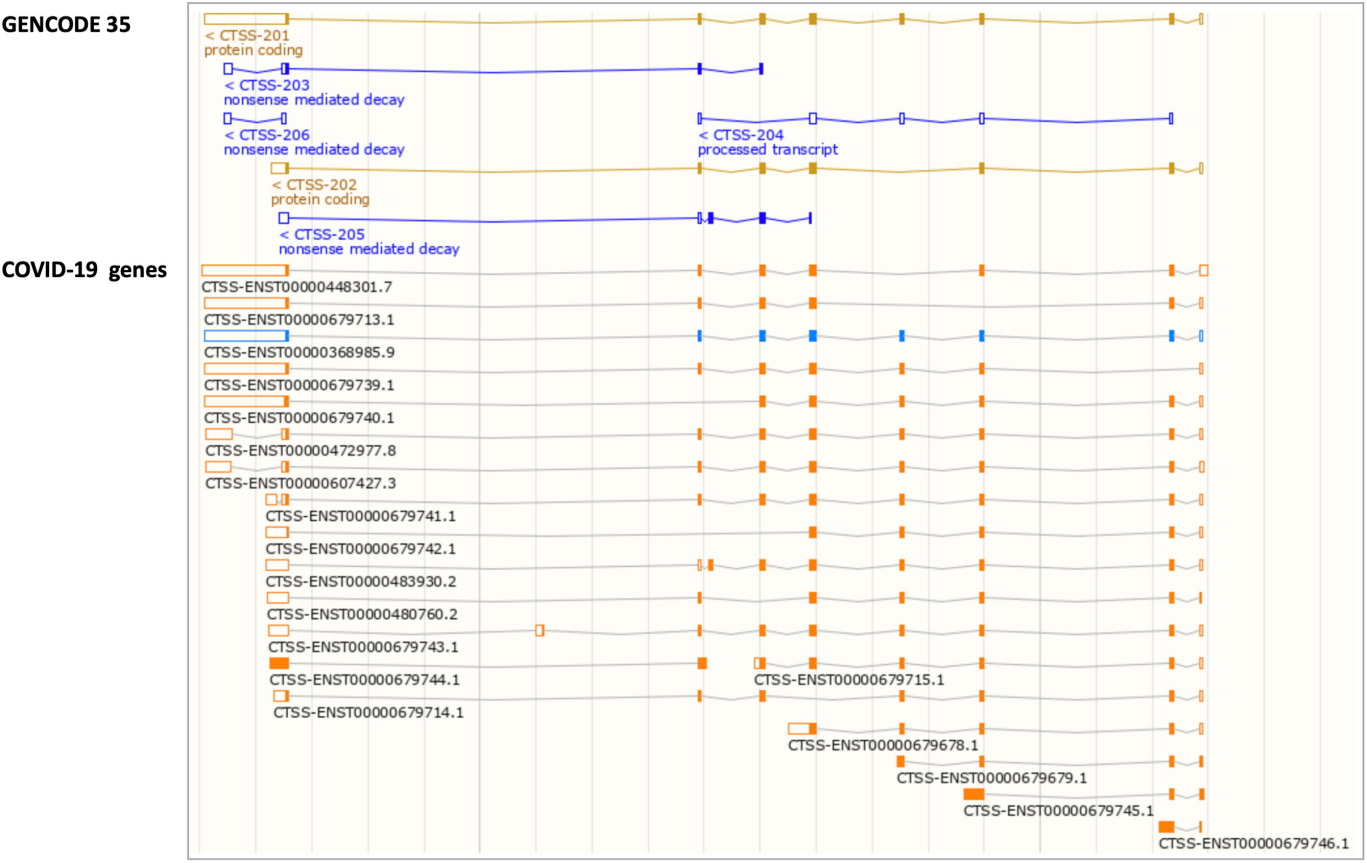
A screenshot from the Ensembl genome browser of the location view for the CTSS gene. The Comprehensive annotation from GENCODE 35 is shown in the upper panel and the updated annotation in the COVID-19 genes trackhub is shown in the lower panel. Transcript models that are unchanged with respect to release Ensembl 101 are coloured blue, whereas new models or pre-existing models that have been modified are shown in orange.

To expedite public access to updated annotation between releases, all annotation changes are made freely available within 24 h via the ‘GENCODE update’ Track Hub, which can be accessed at both the Ensembl and UCSC genome browsers. In the Ensembl browser, the hub has been added to the Track Hub Registry (accessed via the ‘Custom tracks’ section), and can be connected to by searching for ‘GENCODE update’. Alternatively, the data can be added as a custom track in both Ensembl and UCSC browsers (http://ftp.ebi.ac.uk/pub/databases/gencode/update_trackhub/hub.txt). Additionally, a trackhub of updates to genes associated with COVID-19 can be accessed in the same way (http://ftp.ebi.ac.uk/pub/databases/gencode/covid19_trackhub/hub.txt). In the ‘COVID-19 genes’ track data view, transcript models that are unchanged with respect to release GENCODE 35/Ensembl 101 are coloured blue, whereas new models or pre-existing models that have been modified are shown in orange (Figure 3). We also offer BED and gtf files for these annotations.

We have made available the public ‘Synonymous Constraint’ track hub in the UCSC Genome Browser that shows protein-coding regions under synonymous constraint, indicating an overlapping function, and synonymous accelerated regions, indicating a high mutation rate (https://data.broadinstitute.org/compbio1/SynonymousConstraintTracks/trackHub/).

Supported GENCODE annotation is available on the GRCh38 human reference assembly and the GRCm38 mouse reference assembly. Selected human releases are mapped back to the GRCh37 assembly and made available from UCSC and https://www.gencodegenes.org as a service to the community. The resulting mapping are not manually checked and may have errors especially in complicated regions of the human genome. We recommend use of the GRCh38 annotations if possible.

Training about the GENCODE annotation and its use is available from the Ensembl and UCSC training team and user support is available from the Ensembl and UCSC helpdesks.

## CONCLUSION

The GENCODE consortium leverages the best available data, analysis and tools to continually improve the gene annotation of the human and mouse reference genomes. We have developed new methods and workflows to take advantage of the increasing quality and volume of data, and in particular long transcriptomic data, while maintaining the specificity afforded by expert human oversight. We expect our ability to use new data to improve our coverage of novel genes and alternatively spliced transcripts will allow us to move towards a more complete representation of all gene features of known functional classes as we monitor the emergence of new functional features that may require annotation such as alternative translations of known coding genes, non-canonical translations in, for example, lncRNAs and mRNA with multiple functions.
